# Distinct characteristics of BTLA/HVEM axis expression on Tregs and its impact on the expansion and attributes of Tregs in patients with active pulmonary tuberculosis

**DOI:** 10.3389/fcimb.2024.1437207

**Published:** 2024-09-25

**Authors:** Peijun Tang, Xinghua Shen, Jianling Gao, Jianping Zhang, Yanjun Feng, Ji Zhang, Ziyi Huang, Xuefeng Wang

**Affiliations:** ^1^ Department of Biochemistry and Molecular Biology, School of Biology and Basic Medical Sciences, Soochow University, Suzhou, China; ^2^ Department of Tuberculosis, The Fifth People’s Hospital of Suzhou, The Affiliated Infectious Disease Hospital of Soochow University, Suzhou, China; ^3^ Department of Critical Care Medicine, The Fifth People’s Hospital of Suzhou, The Affiliated Infectious Disease Hospital of Soochow University, Suzhou, China; ^4^ Department of Critical Care Medicine, The Fourth Affiliated Hospital of Soochow University, Suzhou, China; ^5^ Department of Ophthalmology, The Second Affiliated Hospital of Suzhou University, Suzhou, China; ^6^ Jiangsu Institute of Clinical Immunology, The First Affiliated Hospital of Soochow University, Suzhou, China; ^7^ Jiangsu Key Laboratory of Clinical Immunology, Soochow University, Suzhou, China; ^8^ Jiangsu Key Laboratory of Gastrointestinal Tumor Immunology, The First Affiliated Hospital of Soochow University, Suzhou, China

**Keywords:** pulmonary tuberculosis, Tregs, BTLA, HVEM, PD-L1, PD-1

## Abstract

**Introduction:**

Pulmonary tuberculosis (PTB) remains one of the deadliest infectious diseases. Understanding PTB immunity is of potential value for exploring immunotherapy for treating chemotherapy-resistant PTB. CD4^+^CD25^+^Foxp3^+^ regulatory T cells (Tregs) are key players that impair immune responses to *Mycobacteria tuberculosis* (MTB). Currently, the intrinsic factors governing Treg expansion and influencing the immunosuppressive attributes of Tregs in PTB patients are far from clear.

**Methods:**

Here, we employed flow cytometry to determine the frequency of Tregs and the expression of B and T lymphocyte attenuator (BTLA) and its ligand, herpesvirus entry mediator (HVEM), on Tregs in patients with active PTB. Furthermore, the expression of conventional T cells and of programmed death-ligand 1 (PD-L1) and programmed death-1 (PD-1) on Tregs in patients with active PTB was determined. We then examined the characteristics of BTLA/HVEM expression and its correlation with Treg frequency and PD-L1 and PD-1 expression on Tregs in PTB patients.

**Results:**

The frequency of Tregs was increased in PTB patients and it had a relevance to PTB progression. Intriguingly, the axis of cosignal molecules, BTLA and HVEM, were both downregulated on the Tregs of PTB patients compared with healthy controls (HCs), which was the opposite of their upregulation on conventional T cells. Unexpectedly, their expression levels were positively correlated with the frequency of Tregs, respectively. These seemingly contradictory results may be interpreted as follows: the downregulation of BTLA and HVEM may alleviate BTLA/HVEM *cis*-interaction-mediated coinhibitory signals pressing on naïve Tregs, helping their activation, while the BTLA/HVEM axis on effector Tregs induces a costimulatory signal, promoting their expansion. Certainly, the mechanism underlying such complex effects remains to be explored. Additionally, PD-L1 and PD-1, regarded as two of the markers characterizing the immunosuppressive attributes and differentiation potential of Tregs, were upregulated on the Tregs of PTB patients. Further analysis revealed that the expression levels of BTLA and HVEM were positively correlated with the frequency of PD-1^+^Tregs and PD-L1^+^Tregs, respectively.

**Conclusion:**

Our study illuminated distinct characteristics of BTLA/HVEM axis expression on Tregs and uncovered its impact on the expansion and attributes of Tregs in patients with active PTB. Therefore, blockade of the BTLA/HVEM axis may be a promising potential pathway to reduce Treg expansion for the improvement of anti-MTB immune responses.

## Introduction

*Mycobacteria tuberculosis* (MTB) infection-caused pulmonary tuberculosis (PTB) remains one of the deadliest chronic human infectious diseases worldwide (Collaborators, 2024; Group, 2024). Due to the emergence of drug-resistant MTB, anti-tuberculosis chemotherapy has been greatly challenged (Dominguez et al., 2023). In addition, the efficacy of *Bacillus Calmette-Guerin* (BCG) has gradually decreased in adults, which poses a challenge for TB prevention (Nadolinskaia et al., 2022; Lee et al., 2023). However, immunotherapy is showing exciting prospects in multiple diseases, including tuberculosis (TB) (La Manna et al., 2020; Gress et al., 2023). Therefore, it is of potential value to uncover the characteristics of the immune response to MTB in order to explore immunotherapy as a treatment for chemotherapy-resistant PTB.

When entering the respiratory tract, MTB is engulfed by alveolar macrophages (AM), but they are inactive at this time, leading to them being ineffective in killing MTB. Fortunately, MTB reactive CD4^+^ T and CD8^+^ T cells can be aroused simultaneously and stimulate AM by releasing interferon-γ (IFN-γ) to ultimately eradicate the engulfed MTB (Sakai et al., 2014; Lin and Flynn, 2015). However, if such T cells are functionally suppressed, MTB will escape from immune responses, causing PTB progression.

Tregs, characterized by the expression of forkhead box protein p3 (Foxp3), possess strong immunosuppressive activity and play a crucial role in preventing autoimmune responses. However, an aberrant increase of Tregs in PTB may impair the anti-MTB immune responses that mainly depend on an interaction between AM and IFN-γ-produced CD4^+^ T cells and CD8^+^ T cells (Kursar et al., 2007; Lin and Flynn, 2015). In fact, accumulating data have demonstrated that the frequency of Tregs is greatly increased (Cardona and Cardona, 2019). In the circumstance of diseases, tumor growth factor-β (TGF-β) and interleukin-2 (IL-2) constitute the main mediators that induce the differentiation of induced Tregs (iTregs) under antigen stimulation. Moreover, multiple costimulatory and other cytokine signals have an impact on the function and expansion of Tregs. For example, tumor necrosis factor-α (TNF-α) and OX40 signals can boost Treg expansion (Baeyens et al., 2015). The TNF-α/TNFR2 pathway can also increase Foxp3 expression and latent TGF-β production in Tregs (Qu et al., 2022). Furthermore, another costimulatory molecule, inducible costimulator (ICOS), has been revealed to limit Treg accumulation and function in visceral adipose tissue (Mittelsteadt et al., 2021). Although programmed death-1 (PD-1) is not essential for the development of Tregs, there are reports that PD-1 can promote or limit peripherally induced Tregs (pTregs), and together with interleukin-10, as well as other markers, constitute an effector phenotype of effector Tregs (Chen et al., 2014; Ellestad et al., 2014; Perry et al., 2022). There is also a report that the deletion of PD-1 destabilizes the lineage identity and metabolic fitness of tumor-infiltrating regulatory T cells (Kim et al., 2023). Additionally, Treg-expressed programmed death-ligand 1 (PD-L1) has also been verified to be an inhibitory factor involved in Treg inhibitory function in our previous study (Feng et al., 2015).

B and T lymphocyte attenuator (BTLA) binding with its ligand, herpesvirus entry mediator (HVEM), exerts an inhibitory effect on T cell activation through its cytoplasmic immunoreceptor tyrosine-based inhibitory motif (ITIM) (Sedy et al., 2005). Interestingly, BTLA can also serve as an activating ligand for HVEM when it presents in *trans* by adjacent cells, thus forming a bidirectional signal pathway between BTLA and HVEM (Sakoda et al., 2011). Unexpectedly, there are reports that BTLA interacts with HVEM in *cis*, a predominant complex formed in naive T cells that blocks HVEM-mediated T cell activation (Cheung et al., 2009; Battin et al., 2022). Moreover, BTLA maintains a costimulatory function mediated by its cytoplasmic Grb2 motif by enhancing the secretion of IL-2 and activation of Src after T cell receptor (TCR) stimulation (Ritthipichai et al., 2017). Hence, the BTLA/HVEM axis possesses a singular ability to not only mediate a bidirectional signal but also provide both costimulatory and coinhibitory signals.

The mechanism regulating the activation and expansion of Tregs not only shares similarities with but also differs from conventional T cells. Some reports have demonstrated that the BTLA/HVEM signal assumes a unique role in Tregs in different contexts. Treg-expressed HVEM, upon binding to BTLA expressed by effector T cells, helps mediate the suppressive functions of Tregs (Tao et al., 2008). BTLA, binding with HVEM, can promote Foxp3 expression in T cells through the upregulation of CD5 (Jones et al., 2016). Administration of an agonistic anti-BTLA antibody can suppress T effector cells but promote Treg expansion in nephrotoxic nephritis (NTN) (Diefenhardt et al., 2023). However, to date, the characteristics of BTLA and HVEM expression on Tregs in PTB patients as well as their influence on Treg expansion and function remain undefined. In the current study, we described the signatures of BTLA and HVEM expression on Tregs in active PTB patients and examined their impact on Treg expansion and function.

## Materials and methods

### Study subjects

Peripheral blood samples from PTB patients and healthy controls (HCs) were collected from The Affiliated Infectious Hospital of Soochow University. Demographic and clinical indicator data of patients with PTB and HCs are presented in **Table 1**. According to the National Diagnostic Criteria for Pulmonary Tuberculosis (WS288-2008), all patients were diagnosed with active PTB. All cases in the study had no other co-infectious diseases such as HIV, HCV, HDV, or HBV, or any other complications, and had not undergone TB treatment before the investigation. Healthy donors from the Physical Examination Department of the Affiliated Hospital for Infectious Diseases of Soochow University had no respiratory infections or other diseases. The study was conducted at The Affiliated Infectious Hospital of Soochow University and approved by the ethics committee of the hospital. Informed consent was provided by the participants.

**Table 1:** Demographic and clinical indicator data of patients with PTB and healthy controls (HCs).

	PTB patients	HCs
Cases	46	40
Age	39.17± 20.33	27.55± 11.45
Sex (Male/Female)	31/15	27/13
Hematogenous tuberculosis	1	/
Secondary tuberculosis	45	/
MTB antibodies (+/-)	10/7	/
MTB culture results (+/-)	19/20	/
Drug resistance (R/S)	6/11	/
CT diagnosis (severe/moderate)	14/16	/

Data are shown as the number or mean± SD; R: drug-resistant, S: sensitive.

### Determination of immune subsets and markers using flow cytometry

The whole blood samples from PTB patients and healthy individuals were collected and human peripheral blood mononuclear cells (PBMCs) were isolated by Ficoll gradient centrifugation. Briefly, the blood samples were diluted with an equal volume of PBS, and then the diluted blood was carefully layered over a density gradient Ficoll-Paque. The samples were centrifuged at 500× g for 30 minutes. After centrifugation, the PBMC layer was collected and the collected PBMCs were washed with PBS by centrifuging at 300×g for 10 minutes. For measurement of the CD4^+^CD25^+^Foxp3^+^ populations that represent Tregs, PBMCs were diluted into 1×10^6^ cells/100 μL. FITC anti-human CD4 (Biolegend, Clone SK3, Cat# 980802) and PE/Cy7 anti-human CD25 (Biolegend, Clone BC96, Cat# 302612) or PE/Cy7 mIgG as an isotype control antibody (Biolegend, Clone MOPC-21, Cat# 981816) were added for staining at 4°C for 30 min. To permeabilize cells to test Foxp3, 1mL of FoxP3/Transcription Factor Permeabilization Buffer working solution (Biolegend, San Diego, CA, USA) was added and incubated for 20 min at 4°C. The cells were then washed with pre-cooled PBS and centrifuged to remove the supernatant. The cells were resuspended in 100μL FoxP3/Transcription Factor Permeabilization Buffer (Biolegend, San Diego, CA, USA) and incubated with Alexa Fluor 647 anti-human Foxp3 (Biolegend, Clone 206D, Cat# 320114) for 30 min at room temperature. After centrifugation and removal of the supernatant twice with PBS, the cells were resuspended in 500 μL FoxP3/Transcription Factor Permeabilization Buffer and analyzed on a flow cytometer. For determination of the BTLA, HVEM, PD-L1, and PD-1 expression on Tregs, FITC anti-human CD4 and PE/Cy7 anti-human CD25 together with PE/Cy7 mouse IgG, PE anti-human HVEM (Biolegend, Clone 122, Cat# 318806), PE anti-human PD-L1 (Clone MIH3, Cat# 374512), PE anti-human PD-1 (Biolegend, Clone EH12.2H7, Cat# 329906), or PE anti-human BTLA (Clone 8H9) that was previously generated in our lab (Wang et al., 2007) were added for staining at 4°C for 30 min. Foxp3 fixation, permeabilization, and staining were performed as described above. For examination of BTLA and HVEM on CD4^+^ T and CD8^+^ T cells, FITC anti-human CD4 or FITC anti-human CD8, together with PE anti-human HVEM or PE anti-human BTLA were added for staining at 4°C for 30 min. All antibodies except for PE anti-human BTLA were purchased from Biolegend (San Diego, CA, USA). All samples were detected using a BD FACSAria (San Jose, CA) and analyzed using FlowJo (San Carlos, CA). The Aria laser/filter setup used in the study was Blue Laser-488nm/(530/30),(575/26), and (780/60) and Red Laser-633nm/(660/20).

### Clinical parameter assays of PTB patients

The demographics of the patients and the healthy individuals are shown in **Table 1**.

#### Determination of antibodies to M. tuberculosis

The measurement of antibodies to M. tuberculosis was performed by using a Diagnostic Kit (Nanjing Potomac Bio-Technology, China). This method was based on a microarray platform, which immobilizes lipoarabinomannan (LAM), 16kD (rTPA16), and 38kD (rTPA38) proteins onto a nitrocellulose membrane. The procedure was conducted according to the standard protocol. The detection of any antibody was considered a positive result (+), and otherwise as a negative result (-).

#### Analysis of pulmonary CT imaging of patients with tuberculosis

All cases of pulmonary tuberculosis were examined by spiral CT imaging at The Affiliated Hospital for Infectious Diseases of Soochow University. A tuberculosis imaging diagnosis was given by the radiologists. The scanning range was from the apex to the base of the lung. In the study, patients with infiltration of the exudative type were categorized into the mild group. Those who had developed cheese-like necrosis and cavitation were categorized into the severe group.

#### Assay of MTB culture

The processed sputum samples are added to BBL MGIL culture tubes containing a mixed additive of growth supplement and heterobacterial inhibitor (PANTA). Then, the inoculated MGIT culture tubes were placed in the BACTEC MGIT-960 Mycobacterium tuberculosis culture instrument for cultivation, and the instrument automatically scanned the tubes to provide negative or positive results for the culture.

#### Drug susceptibility testing for M. tuberculosis

Tuberculosis cultures were performed on Lowenstein-Jensen medium to test resistance to isoniazid, rifampicin, ethambutol, and streptomycin. In this analysis, a colony count >200 was defined as sensitive, a colony count >20 as moderately sensitive, and a colony count ≤20 as drug-resistant. Moderately sensitive cultures were also characterized as drug-resistant.

### Statistical analysis

The data were processed by using GraphPad Prism 9.0 software (GraphPad, San Diego, CA, USA). The results were presented as mean ± standard error of the mean (SEM). A Mann-Whitney test or unpaired *t* test with Welch’s correction was respectively chosen to assess the difference between two different groups according to the distribution of data. A two-tailed *p value* < 0.05 was considered statistically significant.

## Results

### The frequency of CD4^+^CD25^+^Foxp3^+^ Tregs is significantly increased in the peripheral blood of PTB patients

**Figure 1:**
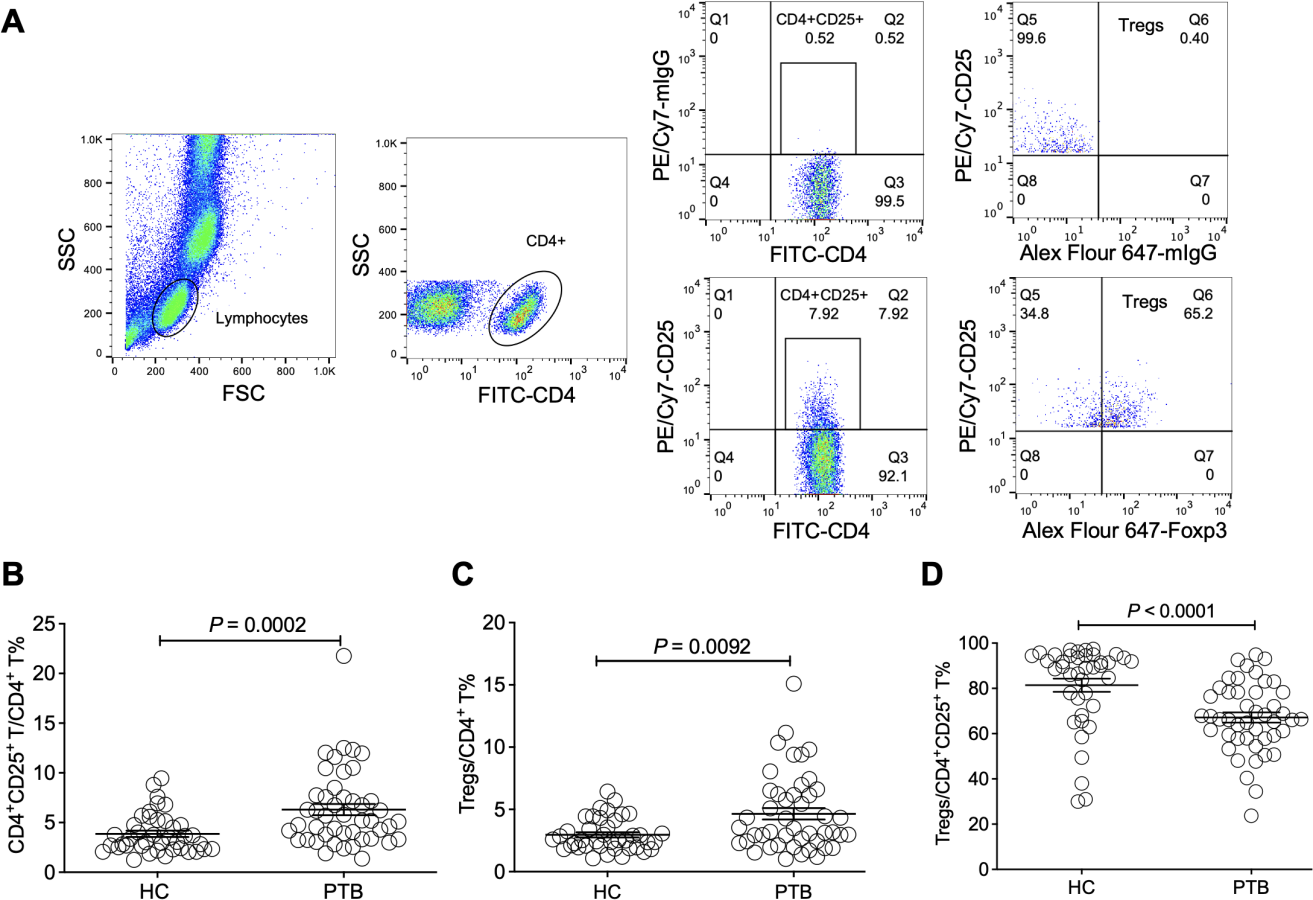
The frequency of Tregs was significantly increased in the peripheral blood of PTB patients. **(A)** The representative diagram shows the determination of CD4^+^CD25^+^ and Treg populations using flow cytometry. **(B)** The percentage of CD4^+^CD25^+^ T cells among the CD4^+^ population in the peripheral blood of the PTB patients was evidently higher than that in the HCs. **(C)** The frequency of Tregs was significantly increased in the peripheral blood of the PTB patients. **(D)** The percentage of Tregs in CD4^+^CD25^+^ T cells was obviously lower in the PTB patients than in the HCs. Tregs, CD4^+^CD25^+^Foxp3^+^ regulatory T cells.

First, we examined the frequency of Tregs in the peripheral blood of PTB patients using flow cytometry (FCM). CD4^+^CD25^+^ T cell and CD4^+^CD25^+^Foxp3^+^ T cell populations were respectively gated as shown in **Figure 1A**. The frequency of CD4^+^CD25^+^ T cells in the PTB patients was evidently higher than that in the HCs (**Figure 1B**), suggesting that the total population, including activated conventional CD4^+^ T cells and Tregs, was upregulated in the peripheral blood of PTB patients since the surface molecule CD25 is one of the hallmarks that reflect T cell activation. As expected, the frequency of CD4^+^CD25^+^Foxp3^+^ T cells was significantly increased in the PTB patients (**Figure 1C**), which was coincident with previous reports (Cardona and Cardona, 2019). Surprisingly, the percentage of CD4^+^CD25^+^Foxp3^+^ T cells occupied in CD4^+^CD25^+^ T cells was significantly downregulated in the PTB patients compared with the HCs (**Figure 1D**).

### The increased frequency of Tregs is related to the progression of pulmonary tuberculosis and the presence of antibodies to *Mycobacteria tuberculosis*

**Figure 2:**
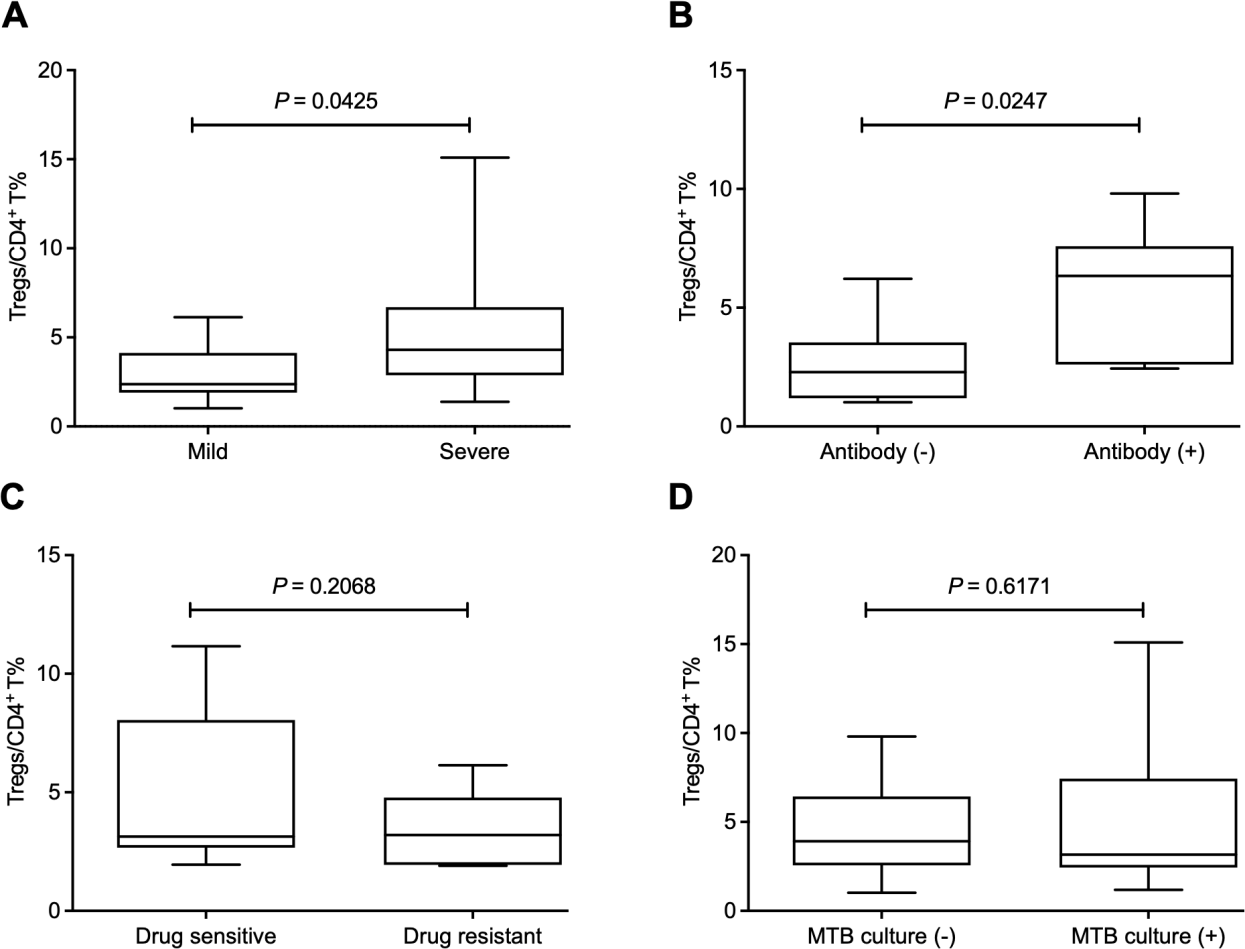
The increased frequency of Tregs is related to the progression of pulmonary tuberculosis and the presence of antibodies to *Mycobacteria tuberculosis.* **(A)** The mild patients had a lower Treg frequency than the severe patients. **(B)** The frequency of Tregs was significantly higher in positive patients for MTB antibodies than those with negative results. **(C, D)** The frequency of Tregs showed no difference between drug-sensitive and resistant patients **(C)** or between MTB culture-positive and negative patients **(D)**. Tregs, CD4^+^CD25^+^Foxp3^+^ regulatory T cells.

Since Tregs have an immunosuppressive effect on anti-MTB immune responses, we further explored the clinical significance of the increased frequency of Tregs. We defined mild and severe PTB patients according to CT imaging. The results showed that the mild patients had a lower Treg frequency than the severe patients (**Figure 2A**). Additionally, the relationship between Treg frequency and the presence of antibodies to the lipoarabinomannan (LAM), 38 kDa, or 16 kDa antigens of MTB was investigated. Our data showed that the frequency of Tregs was significantly higher in patients positive for MTB antibodies than those with negative results (**Figure 2B**). However, there was no difference in Treg frequency between drug-sensitive and resistant patients or between MTB-culture-positive and negative patients (**Figures 2C, D**).

### BTLA and HVEM were downregulated on Tregs, which is the opposite of their upregulation on conventional T cells

**Figure 3:**
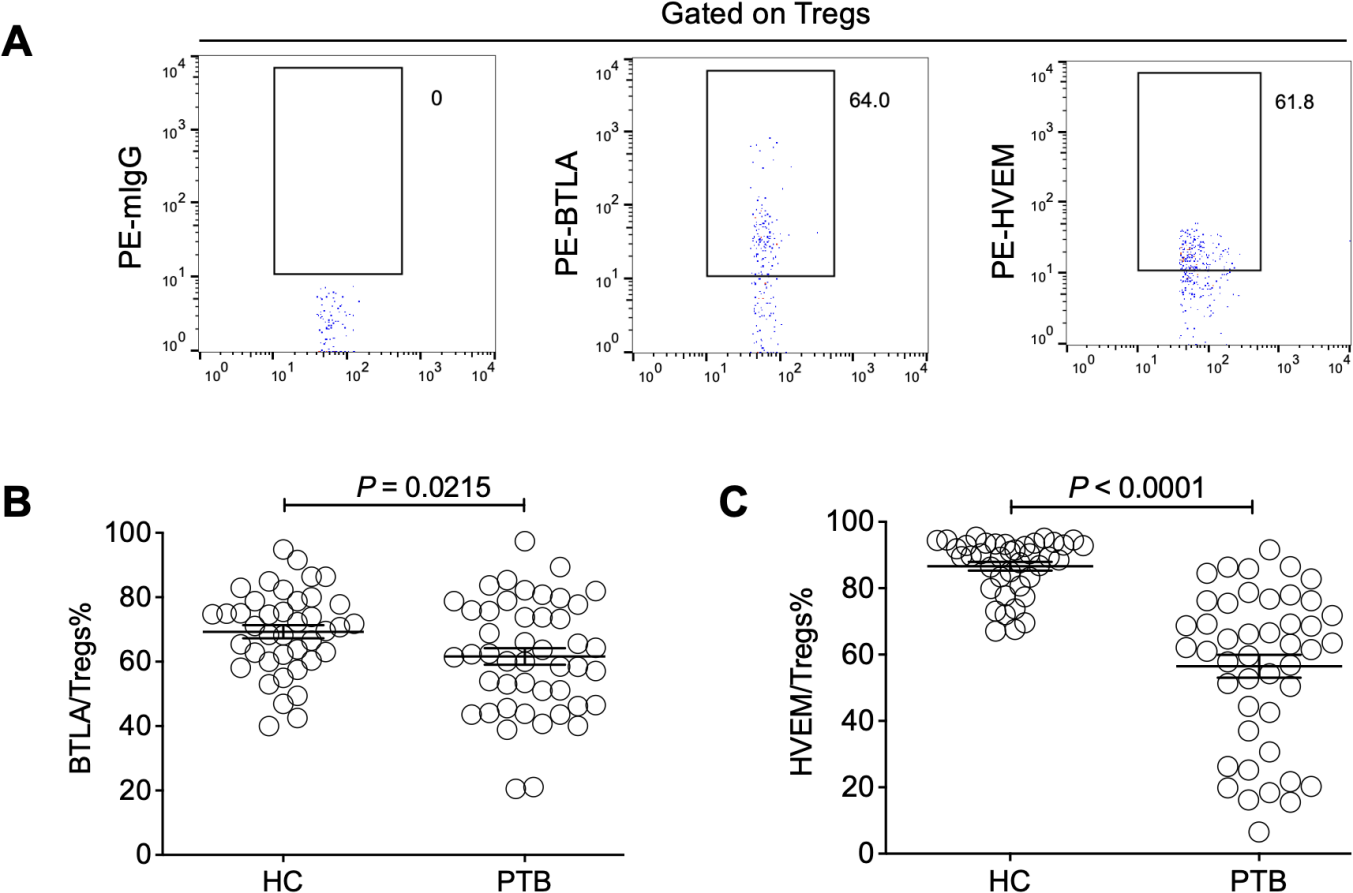
The expression levels of BTLA and HVEM on Tregs were downregulated in the PTB patients. **(A)** The gating strategy for measuring BTLA and HVEM expression on Tregs is shown. **(B, C)** Both BTLA and HVEM were downregulated on the Tregs of the PTB patients compared with the HCs.

**Figure 4:**
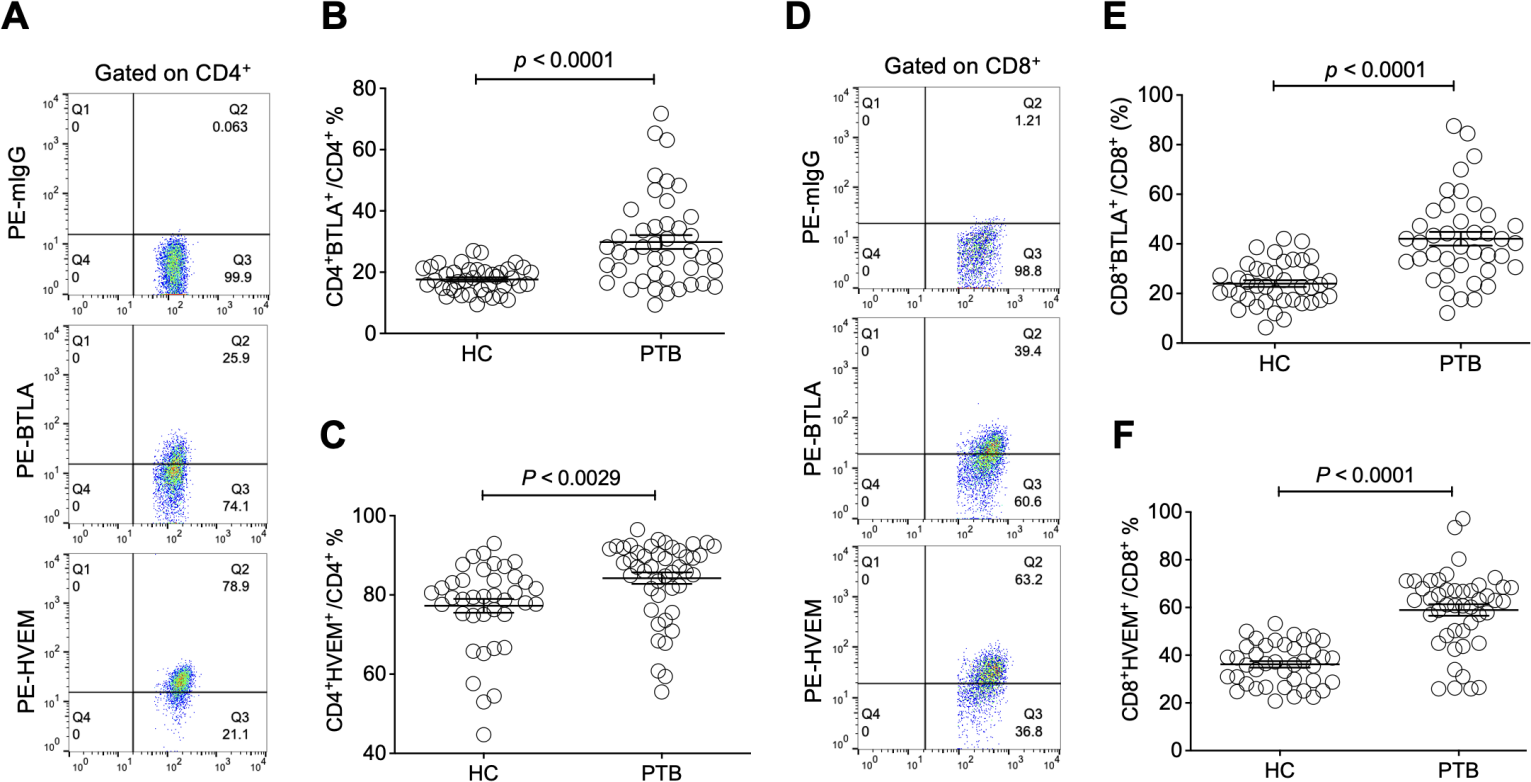
The expression levels of BTLA and HVEM on CD4^+^ T and CD8^+^ T cells were upregulated in the PTB patients. **(A)** The gating strategy for measuring BTLA and HVEM expression on CD4^+^ T cells is shown. **(B, C)** The expression levels of BTLA and HVEM on CD4^+^ T cells were upregulated in the PTB patients compared with the HCs. **(D)** The gating strategy for measuring BTLA and HVEM expression on CD4^+^ T cells is shown. **(E, F)** The expression levels of BTLA and HVEM on CD8+ T cells were upregulated in the PTB patients compared with the HCs.

Moreover, we sought to investigate the influence of Treg-expressed BTLA and HVEM on Treg expansion in PTB patients. Through determination by FCM (**Figure 3A**), our data demonstrated that both BTLA and HVEM were downregulated on the Tregs of PTB patients (**Figures 3B, C**). Previously, we demonstrated that BTLA was upregulated on conventional T cells and the increased frequency of BTLA^+^CD4^+^ T and BTLA^+^CD8^+^ T cells was closely associated with PTB progression (Shen et al., 2019). Furthermore, we examined the expression characteristics of BTLA and HVEM on conventional T cells in the current samples. As expected, BTLA was upregulated. Simultaneously, HVEM was also upregulated on CD4^+^ T and CD8^+^ T cells (**Figures 4A–F**). Therefore, the expression pattern of BTLA and HVEM on Tregs is different from that on conventional T cells.

### The expression levels of BTLA and HVEM were positively correlated with Treg frequency

**Figure 5:**
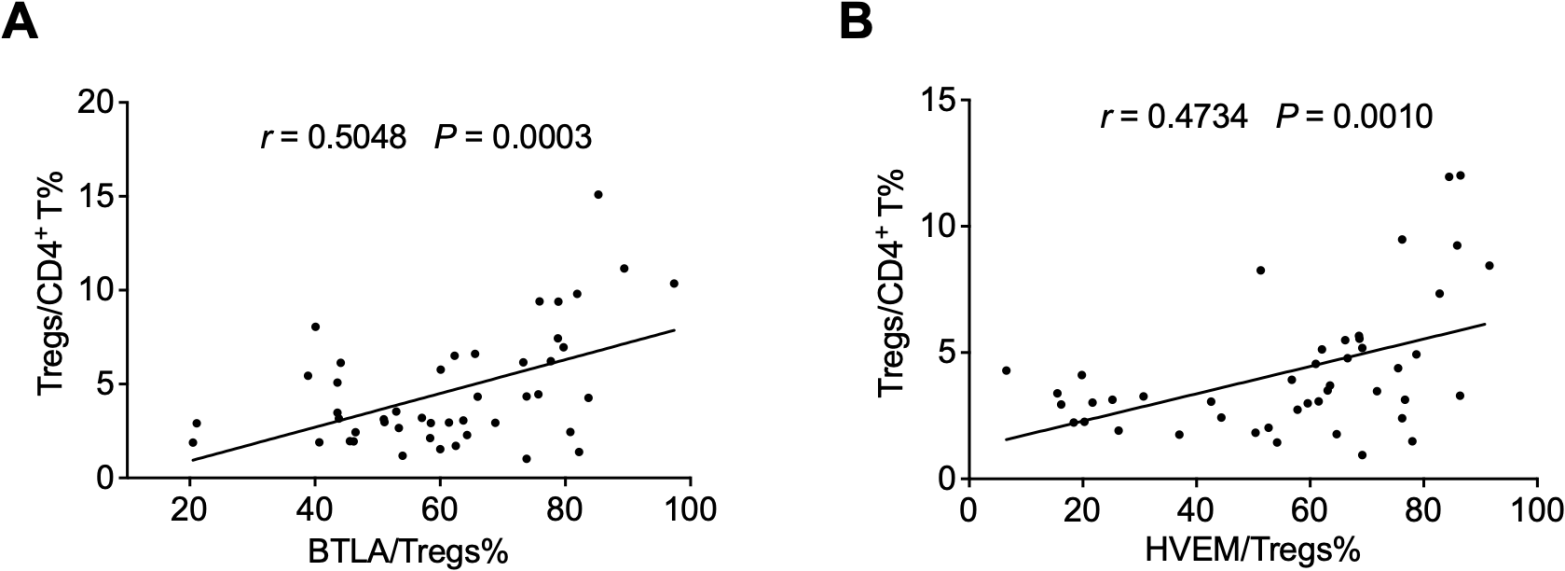
The expression levels of BTLA and HVEM were positively correlated with Treg frequency. **(A)** The expression levels of BTLA were positively correlated with the frequency of Tregs. **(B)** The expression levels of HVEM were positively correlated with the frequency of Tregs. Tregs, CD4^+^CD25^+^Foxp3^+^ regulatory T cells.

Since the downregulation of BTLA and HVEM expression may be beneficial for Treg expansion, we sought to investigate the association between BTLA and HVEM expression and Treg frequency. Unexpectedly, the expression levels of BTLA and HVEM were positively correlated with the frequency of Tregs, respectively (**Figures 5A, B**).

### The expression of BTLA and HVEM was closely associated with Treg-expressed PD-L1 in PTB patients

**Figure 6:**
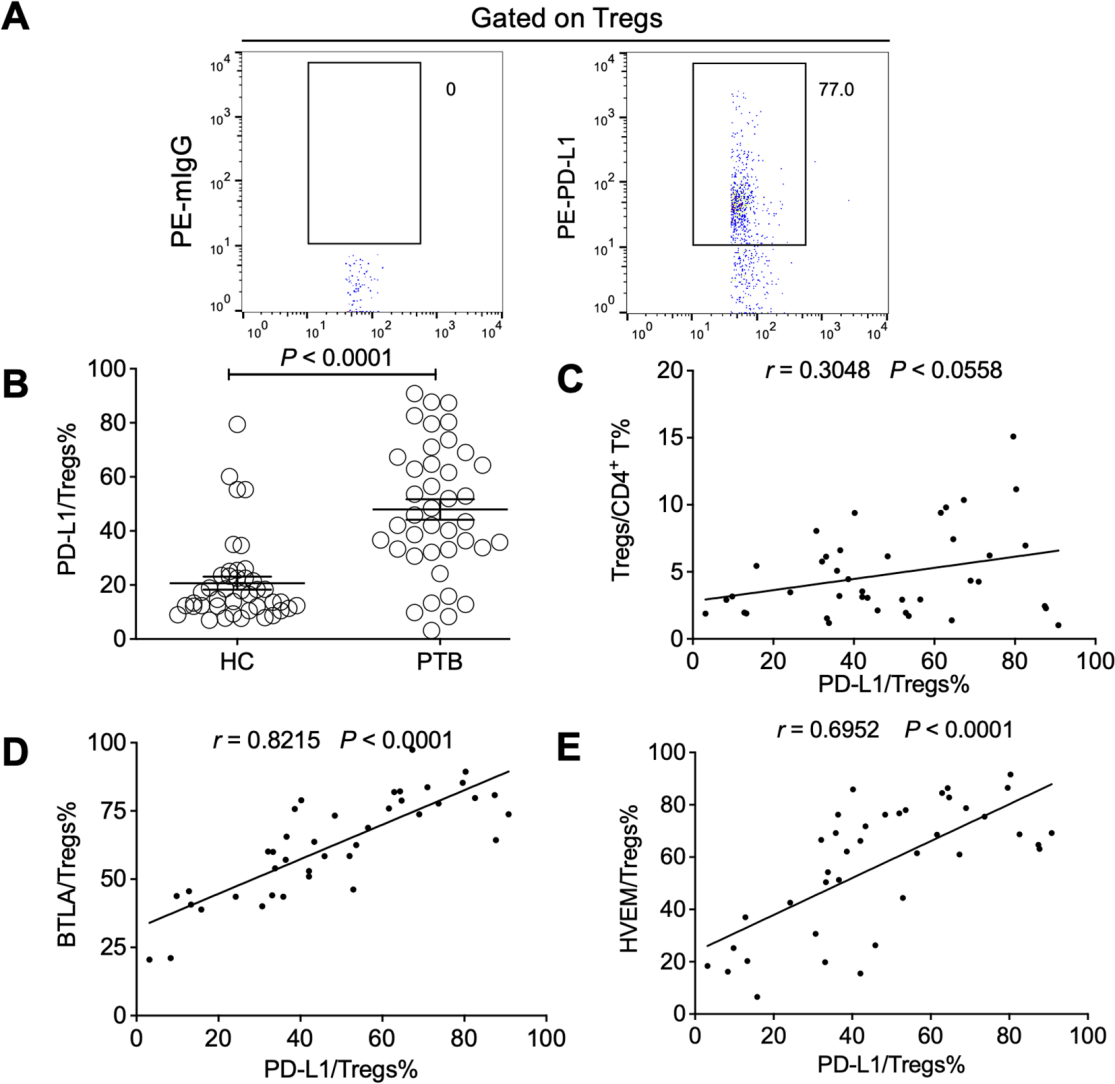
The expression levels of BTLA and HVEM were positively correlated with Treg-expressed PD-L1 in the PTB patients. **(A)** The gating strategy for measuring PD-L1 in Tregs from PTB patients is presented. **(B)** PD-L1 was upregulated on the Tregs of the PTB patients compared with the HCs. **(C)** The association between the expression levels of PD-L1 on Tregs and Treg frequency was not significant. **(D, E)** PD-L1 expression on Tregs was positively correlated with the expression of BTLA and HVEM, respectively. Tregs, CD4^+^CD25^+^Foxp3^+^ regulatory T cells.

There was a report that Tregs in PTB mainly rely on a cell-cell contact-dependent mechanism and not on suppressive cytokines such as TGF-β, IL-10, and IL-35 to exert their inhibitory effect (Chen et al., 2007). Our previous findings have demonstrated that the progression of chronic hepatitis B virus (HBV) infection was closely associated with an increase of Treg-expressed PD-L1 (Xu et al., 2014), probably through restraining HBV immunity by engaging with PD-1 expressed on effector T cells. Thus, we examined PD-L1 expression and its relationship with the expression of BTLA and HVEM on the Tregs of PTB patients. As expected, PD-L1 was found to be significantly upregulated in PTB patients but not associated with Treg expansion (**Figures 6A–C**). However, interestingly, PD-L1 expression on Tregs was closely and positively correlated with the expression levels of both BTLA and HVEM, respectively (**Figures 6D, E**), indicating that the BTLA/HVEM axis might present in a *trans*-interaction manner and that these axis-expanded Tregs have significantly immunosuppressive activity as reflected by PD-L1.

### The expression of BTLA and HVEM had a close relationship with Treg-expressed PD-1 in patients with pulmonary tuberculosis

**Figure 7:**
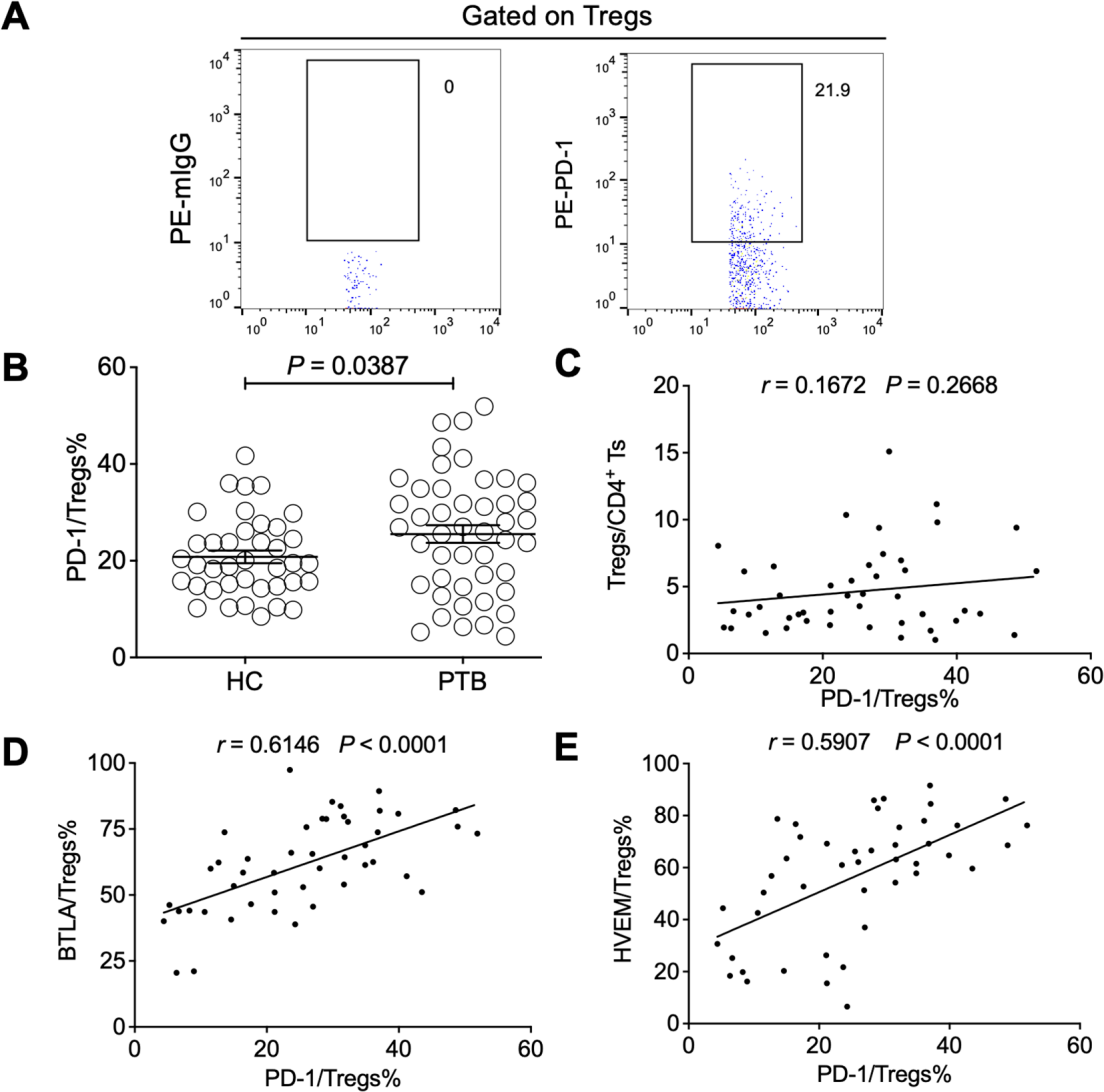
The expression levels of BTLA and HVEM were positively correlated with Treg-expressed PD-1 in the PTB patients. **(A)** The gating strategy for measuring PD-1 in Tregs from PTB patients is shown. **(B)**. PD-1 was upregulated on the Tregs of the PTB patients compared with the HCs. **(C)** The expression levels of PD-1 on Tregs were not associated with Treg frequency in the PTB patients. **(D, E)** PD-1 expression on Tregs was positively correlated with the expression of BTLA and HVEM, respectively. Tregs, CD4^+^CD25^+^Foxp3^+^ regulatory T cells.

PD-1 can negatively regulate Treg expansion at homeostasis and during inflammatory processes (Perry et al., 2022), while a blockade of PD-1 signals significantly induces PD-1^+^ Treg cell activation in patients with tumors (van Gulijk et al., 2023). However, a PD-1 signal is also reported to drive Treg cell expansion, causing tumor evasion, and PD-1^+^ Treg cells are considered a potential prognostic predictor in patients with tumors (Dong et al., 2020). In this study, we found that PD-1 was upregulated on the Tregs of PTB patients but it was not relevant to Treg expansion (**Figures 7A–C**). Intriguingly, PD-1 expression on Tregs was positively correlated with the expression levels of both BTLA and HVEM, respectively (**Figures 7D, E**), further demonstrating that the BTLA/HVEM axis presented on effector Tregs in a *trans*-interaction manner may enhance the function of Tregs in tuberculosis.

## Discussion

MTB remains one of the top microbial killers, causing approximately 1.3 million deaths (Singh, 2024). More than 90% of MTB-infected individuals never develop active PTB, suggesting that the immune system is capable of controlling MTB infection. However, immune responses generally fail to achieve complete clearance of MTB. One of the reasons for this may be that the immune subsets responsible for eliminating MTB are functionally suppressed by inhibitory factors (Hmama et al., 2015). Regulatory T subsets including CD4^+^CD25^+^Foxp3^+^ T and CD8^+^CD28^-^ T cells are obviously increased in PTB patients and suppress MTB immunity (Sun et al., 2012; Bertolini et al., 2018; Yu et al., 2022), of which the former is the dominant immunosuppressive subset in PTB as well as other infectious diseases.

An abnormal increase in Tregs may contribute to the suppression of Th1-type immune responses against MTB, leading to PTB progression (Jaron et al., 2008; Jasenosky et al., 2015). Our data showed that an increase in Tregs was closely related to the presence of anti-MTB antibodies and PTB progression, indicating that Tregs may be expanded in a feedback manner along with an enhancement of anti-MTB immune responses, reflected by the appearance of antibodies to MTB, and an increase in Tregs impairs MTB immunity, causing PTB progression. However, the frequency of Tregs is not related to drug resistance and MTB sputum culture results suggested that the variation in immune responses may not be obviously influenced by MTB strains associated with drug resistance and the manifestation of MTB in the respiratory tract. As for the finding that the frequency of Tregs in CD4^+^CD25^+^ T cells in PTB patients was lower than that in HCs, it can be interpreted that the number of Tregs in the HCs was relatively stable for maintaining immune homeostasis and the number of activated conventional CD4^+^ T cells was very low, while in the PTB patients, a comparable number of conventional CD4^+^ T cells was activated. These findings are in accordance with previous reports and a decrease in Treg frequency is a valuable indicator for tuberculosis treatment (Jaron et al., 2008; Wu et al., 2010). In our study, we gated lymphocytes through the SSC/FSC gating strategy, causing a risk of CD4^+^ monocyte contamination, which is a limitation in our study.

Although our results and those of other investigations clearly showed that Tregs were significantly expanded in PTB patients, the mechanisms driving Treg expansion remain to be explored. Here, for the first time, we found that both BTLA and HVEM were downregulated on the Tregs of PTB patients compared with HCs, in contrast to the fact that they are evidently upregulated on conventional CD4^+^ T and CD8^+^ T cells in tuberculosis as well as other infectious diseases (Shen et al., 2019; Song et al., 2022). The downregulation of BTLA/HVEM expression may lower the threshold of Treg activation, helping Treg expansion and leading to tuberculosis progression. Intriguingly, it seems contradictory that the expression levels of BTLA and HVEM are positively correlated with the frequency of Tregs in tuberculosis. The possible reason may be that the downregulation of BTLA and HVEM may alleviate BTLA/HVEM *cis*-interaction-mediated coinhibitory signals pressing on naïve Tregs, helping their activation, while the BTLA/HVEM axis on effector Tregs induces a costimulatory signal, promoting their expansion through a BTLA/HVEM reverse interaction and/or the BTLA intracellular Grb2 domain-mediated pathway (Demerle et al., 2023). As for the opposite effect of BTLA and HVEM on Tregs and conventional T cells, the mechanism may lie in the different intracellular contexts of effector Tregs and conventional T cells, which may activate BTLA-Grb2 or the BTLA-ITIM motif. However, these interpretations require further investigation by experiments.

A previous report found that the immunosuppressive effects of Tregs in MTB infection might not mainly depend on secreting TGF-β and IL-10, but probably rested on a cell-cell contact-dependent mechanism (Chen et al., 2007). Treg-expressed PD-L1 was proven to be involved in such cell-cell contact-mediated immunosuppression. For example, PD-L1 was reported to contribute to Treg-mediated protection in murine crescentic glomerulonephritis (Neumann et al., 2019). PD-L1 on Tregs was identified to be associated with the progression of hepatitis B virus infection (Feng et al., 2015). In view of these findings, Treg-expressed PD-L1 may be considered one of the indicators that characterize the inhibitory function of Tregs. In this study, our data demonstrated that PD-L1 was also significantly upregulated on the Tregs of PTB patients. Since the *trans*-interaction of BTLA/HVEM may support Treg expansion, we speculated whether the levels of BTLA and HVEM expression have a relationship with the frequency of PD-L1^+^ Tregs. Unexpectedly, both of the frequencies of BTLA^+^ Tregs and HVEM^+^ Tregs were discovered to be positively correlated with that of PD-L1^+^ Tregs, thus indicating that the BTLA/HVEM axis probably presents in a *trans*-interaction manner and is helpful for the expansion of PD-L1^+^ Tregs. Notwithstanding, while such a deduction is reasonable regarding the impact of BTLA and HVEM expression on Treg amplification, further experimental exploration is required.

Interestingly, there are reports that TB-driven BTLA upregulation in dendritic cells (DCs) impairs their function and promotes Foxp3^+^ Tregs (Cai et al., 2019; Zhang et al., 2020). In comparison, BTLA is downregulated in Foxp3^+^ Tregs but causes a similar outcome in that Tregs are expanded, leading to PTB progression. However, the underlying mechanism of TB-driven BTLA upregulation in DCs but downregulation in Tregs requires further study.

Meanwhile, the PD-1 signal has an important influence on Treg activation and expansion. Our data demonstrated that different from BTLA and HVEM, PD-1 was significantly upregulated on Tregs in tuberculosis. As a coinhibitory receptor, PD-1 can negatively regulate Treg expansion at homeostasis and during inflammatory processes by binding with myeloid cell-expressed PD-L1 (Perry et al., 2022). In patients with lung cancer and mesothelioma, a blockade of PD-1 signals significantly induces PD-1^+^ Treg cell activation (van Gulijk et al., 2023). Here, PD-1 was upregulated on Tregs of PTB patients and probably acts as a sensor to tune the pool of effector Treg cells, helping to maintain the function of MTB-reactive T cells and macrophages. Nevertheless, there are reports that PD-1 is critical for the extrathymic differentiation of peripherally induced Treg (iTreg) cells *in vivo* and it is dispensable for natural thymic Treg cell (nTreg) development and suppressive function (Chen et al., 2014; Kos et al., 2022). PD-L1 expression by acute myeloid leukemia (AML) cells may directly drive Treg cell expansion as a mechanism of immune evasion and the frequency of PD-1^+^ Treg cells is a potential prognostic predictor in patients with AML (Dong et al., 2020). These seemingly contradictory results demonstrate the complex role of PD-1 signals in modifying iTreg differentiation and expansion. It is perhaps the contexts of the various diseases or experiments that endow PD-1 with distinct roles in iTregs. Therefore, PD-1 is also considered a marker depicting the effector phenotype of effector Tregs. In this study, we did not find a significant relationship between PD-1 expression and the frequency of Tregs in PTB patients. However, PD-1 expression was positively correlated with BTLA and HVEM expression on Tregs, respectively, further suggesting that the BTLA/HVEM axis in a *trans* model promotes the function and expansion of Tregs in tuberculosis.

**Figure 8:**
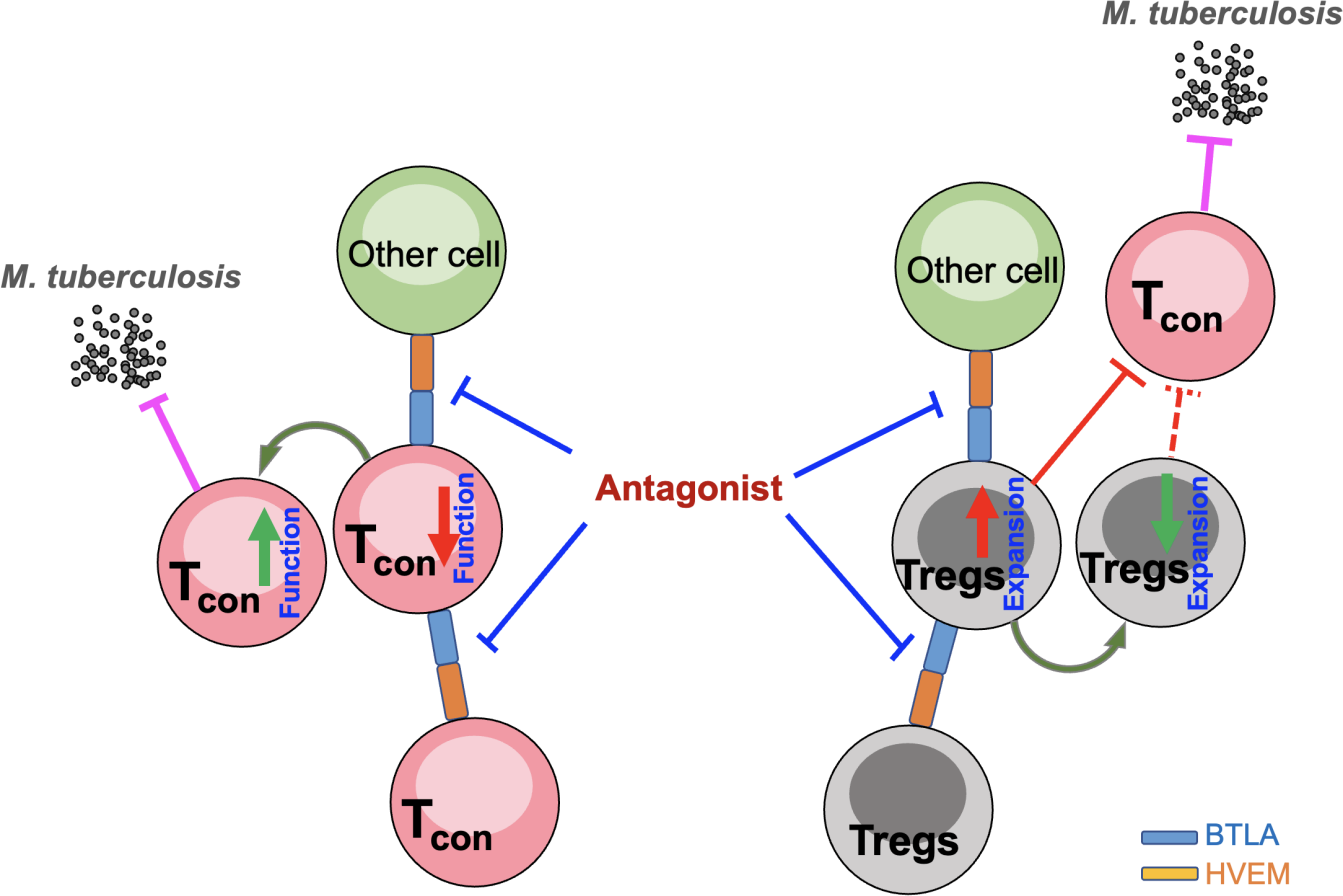
Hypothesis of the effect of a blockade of the BTLA/HVEM axis on impeding Treg expansion and simultaneously enhancing conventional T cell function. Tcon, conventional T cells; Tregs, CD4^+^CD25^+^Foxp3^+^ regulatory T cells.

Immunotherapy based on the blockade of immune checkpoints such as PD-1 or PD-L1 has achieved significant success in tumor immunotherapy. However, a PD-1 blockade may cause an increase in Treg cells, leading to disease progression (Kumagai et al., 2020). In fact, a PD-1 blockade exacerbated MTB infection in an animal model (Kauffman et al., 2021). Intriguingly, the findings in the current study together with our previous report demonstrated that BTLA/HVEM axis expression was positively correlated with Treg expansion and might negatively impact the function of conventional T cells in PTB. Therefore, a blockade of the BTLA/HVEM axis may impede Treg expansion and simultaneously enhance conventional T cell functioning, making it a more valuable potential target than PD-1 for PTB immunotherapy (**Figure 8**).

## Conclusion

In summary, our study for the first time demonstrated the distinct characteristics of BTLA/HVEM axis expression on Tregs that were different from those on conventional T cells in PTB patients and revealed that the unique signatures of their expression were associated with Treg expansion. At the same time, we substantiated that the expression of BTLA and HVEM on Tregs was closely associated with Treg attributes that are reflected by PD-1 and PD-L1. A blockade of the BTLA/HVEM axis may impede Treg expansion and simultaneously enhance conventional T cell function, making it a more valuable potential target than PD-1 for PTB immunotherapy.

## Data Availability

The original contributions presented in the study are included in the article/supplementary material. Further inquiries can be directed to the corresponding authors.
